# Identification of microRNAs in Response to Drought in Common Wild Rice (*Oryza rufipogon* Griff.) Shoots and Roots

**DOI:** 10.1371/journal.pone.0170330

**Published:** 2017-01-20

**Authors:** Jing-wen Zhang, Yan Long, Man-de Xue, Xing-guo Xiao, Xin-wu Pei

**Affiliations:** 1 Biotechnology Research Institute, Chinese Academy of Agricultural Sciences, Beijing, China; 2 State Key-Lab of Plant Physiology and Biochemistry, College of Biological Sciences, China Agriculture University, Beijing, China; Kunming University of Science and Technology, CHINA

## Abstract

**Background:**

Drought is the most important factor that limits rice production in drought-prone environments. Plant microRNAs (miRNAs) are involved in biotic and abiotic stress responses. Common wild rice (*Oryza rufipogon* Griff.) contains abundant drought-resistant genes, which provide an opportunity to explore these excellent resources as contributors to improve rice resistance, productivity, and quality.

**Results:**

In this study, we constructed four small RNA libraries, called CL and CR from PEG6000-free samples and DL and DR from PEG6000-treated samples, where ‘R’ indicates the root tissue and ‘L’ indicates the shoot tissue. A total of 200 miRNAs were identified to be differentially expressed under the drought-treated conditions (16% PEG6000 for 24 h), and the changes in the miRNA expression profile of the shoot were distinct from those of the root. At the miRNA level, 77 known miRNAs, which belong to 23 families, including 40 up-regulated and 37 down-regulated in the shoot, and 85 known miRNAs in 46 families, including 65 up-regulated and 20 down-regulated in the root, were identified as differentially expressed. In addition, we predicted 26 new miRNA candidates from the shoot and 43 from the root that were differentially expressed during the drought stress. The quantitative real-time PCR analysis results were consistent with high-throughput sequencing data. Moreover, 88 miRNAs that were differentially-expressed were predicted to match with 197 targets for drought-stress.

**Conclusion:**

Our results suggest that the miRNAs of *O*. *rufipogon* are responsive to drought stress. The differentially expressed miRNAs that are tissue-specific under drought conditions could play different roles in the regulation of the auxin pathway, the flowering pathway, the drought pathway, and lateral root formation. Thus, the present study provides an account of tissue-specific miRNAs that are involved in the drought adaption of *O*. *rufipogon*.

## Introduction

To respond to changes in the environment, plants have developed remarkable capabilities to adapt to extreme environments, such as high temperature, drought, and salt stress [1]. Abiotic stress is an important factor that limits plants geographic distribution and yield. Therefore, one central goal is the improvement of stress tolerance in crops.

Common wild rice (CWR) is the ancestor of cultivated rice (*Oryza sativa* L.), and it has great genetic diversity and has been researched extensively. During the entire developmental process of rice, early drought causes transplanting delays, seed germination, and growth of seedlings, resulting in harvest failure. Drought in the reproductive period leads to different degrees of spikes in grain sterility and poor grain filling [2]. In one study of drought tolerance in CWR, Hu et al. examined drought resistance for 3 years [3]. They demonstrated that CWR has more roots and a better survival rate compared with cultivated rice, indicating that CWR has developed a water delivery system. Four groups of Dongxiang CWR collected from three original habitat populations were compared with 15 rice cultivars for drought resistance in the seedling stage. The drought resistance was measured using index traits, including root length, stem length, fresh root weight, dry root weight, and drought resistance index, and they found that Dongxiang CWR is more tolerant to water shortage than cultivated rice [4]. In addition, a hybrid population of cultivars rice and CWR was constructed to analyze the quantitative trait locus (QTL). Subsequently, 12 drought resistance-related QTLs were mapped. Furthermore, a drought tolerant introgression line, IL23, that contained two QTLs, *qSDT2-1* and *qSDT12-2*, was associated with drought tolerance and exhibited strong drought resistance [5]. Liu et al. demonstrated that CWR could be used to modify the stomatal conductance and membrane stability, which are related to drought resistance in cultivars rice [6]. Based on high-throughput sequencing, there were 901 contig responses to drought stress [7]. Therefore, there are abundant drought resistant-gene resources in CWR, providing excellent resources that can contribute to increasing the rice resistance, productivity, and quality [8].

MicroRNAs (miRNAs) are a class of non-coding, endogenous small RNAs that have essential roles in plant development, phase transition, and environment stress [9–10]. The majority of miRNAs can pair with multiple targets. Plant miRNAs bind target mRNAs with a very small number of mismatches or small gaps to regulate the target mRNAs expression via either mRNA degradation or translational inhibition [11–12]. It is important to cleave targets by perfect pairing at the 5’ end at the 9^th^to 11^th^position of complementarity [9,13]. Plant miRNAs are involved in biotic and abiotic stress responses [14–15], and 14 differentially expressed miRNAs respond tosalinity, drought, and cold stress. In addition, miR168, miR171, and miR396 are involved in the three stresses, as determined by microarray data [16]. Under conditions of Cadmium(Cd) stress, 141 known miRNAs and 39 miRNAs express in the root and shoot, including miR156, miR159, miR168, miR169, miR171, miR369, miR529, miR1433, miR1440, etc. [17]. In rice, miR164 targets 6 *OMTN*, which are typical transcript factors and respond to abiotic stress. Drought at the reproductive stage and over-expression of *OMTN2*, *OMTN3*, *OMTN4* and *OMTN6* decrease the drought tolerance of rice. Simultaneously, genes that are involved in stress, development, and metabolism are down-regulated in *OMTN* transgenic lines during drought stress, which have the opposite expression pattern in the wild type during drought stress, indicating that miR164 negatively regulates *OMTN* during drought stress [18]. In *Arabidopsis thaliana*, miR169 regulates *NF-YA2* targets at the post-transcriptional level and down-regulates the response to drought tolerance. Furthermore, miR169 targets *NF-YA2* to control root architecture [19]. Recently, much research onmiRNAs function has been reported in cultivars rice. Thus far, 713 mature miRNAs have been identified in miRBase (Release 21: June 2014). There are several reports of miRNAs in CWR. Wang et al. constructed miRNAs libraries for CWR and cultivars rice, and 259 specific-miRNAs were identified in the *O*. *rufipogon* genome, suggesting a loss of these miRNAs in cultivated rice [20]. High-throughput sequencing technology has identified miRNAs from Dongxiang wild rice, and 33 differentially expressed miRNAs responded to drought stress, including miR164c, miR319b, miR444, miR166h-5p, miR172d-5p, miR167h-3p, miR160f-5p, etc. [21]. Moreover, *Oryza coarctata* grows normally in saline water, and 338 known miRNAs and 95 novel miRNAs were found to respond to salt stress [22]. In 2013, we also identified flowering-related miRNAs in CWR [23].

Cultivars rice (*Oryza sativa*) is thought to be domesticated from CWR. During domestication, rice undergoes significant phenotypic changes in grain size, color, shattering, seed dormancy, and tillering [24]. Currently, research on rice miRNAs focuses on responding to drought [25], salinity [26] and, Pi starvation [27], ABA [28], Cd [17], and rice stripe virus [29]stress, and these studies have made progress. However, there are fewer reports on the exploration of miRNAs to drought responsiveness in CWR. The aims of this study was to identify the drought responsiveness of miRNAs in CWR shoot and root tissues to provide a molecular understand for the response of rice to drought.

## Materials and Methods

### Plant culture and drought stress

Seeds of CWR were obtained from Gaozhou,Guangdong Province, China, and were approved by the supervision department of Guangdong wild rice protection. These seeds were surface-sterilized and were pre-germinated in tap water at 37°C for 3 d in the dark. Germinated seeds were then transferred into a plastic pot and grown on Yoshida culture solution [30] in a plant growth chamber at a day/night temperature of 30°C/25°C (8 h day/16 h night). The relative humidity of the plant growth chamber was 60%-70%. We adjusted the pH of the solution to 5.5, and the solution was renewed every 3 d.

Then, 3-week-old seedlings were subjected to drought stress. Half of the seedlings were treated with 16% PEG6000 for 24h, whereas the other half of the seedlings continuously remained in the culture solution as a control. Roots and shoots were harvested separately and were immediately frozen in liquid nitrogen and stored at −80°C.

### Small RNA isolation and sequencing

The total RNA from the PEG6000-free and PEG6000-treated groups was extracted using TRIzol reagent (Invitrogen,USA) according to the manufacturer’s instructions. Gel electrophoresis was used to detect the quality of RNA, and the concentration of RNA was measured using a NanoDrop 2000 (Thermo Scientific, USA).

After testing, RNA libraries were constructed using a small RNA Sample Pre Kit (NEB, USA). Briefly, the 5’ and 3’ adaptors were ligated sequentially to the small RNAs using T4 RNA Ligase 1 and T4 RNA Ligase 2. The ligated adaptors with the sRNAs were reverse transcribed, and PCR was subsequently performed to construct the final libraries. Then, the prepared libraries were sequenced using an Illumina HiSeq 2000 platform (Biomarker, Beijing). The sequencing profiles have deposited into NCBI SRA, and the accession numbers of the data sets as SRX2037183.

### Data analysis

Data cleaning was performed on 50-nt sequencing tags. To ensure the accuracy of the analysis, the quality of the raw data had to be controlled. The quality control standard for the original sequence was as follows: (1) removal of low quality reads; (2) removal of unknown base N (N represents unrecognized base)-content reads that were greater than 10%; (3) removal of reads without 3′- adaptors; (4) removal of reads without an insert tag; (5) removal of reads without 5′- adaptors; and (6) removal of reads < 18 nt and >30 nt in length. The final clean reads that were 18–30 nt long were used for further analysis. Sequences matching the rRNA, tRNA, snRNA, and snoRNA and repeats of the sequence tags were removed based on Silva (https://www.arb-silva.de/), GtRNAdb (http://gtrnadb.ucsc.edu/), Repbase (http://www.girinst.org/), and the Rfam database (http://rfam.sanger.ac.uk/), and unannotated reads were obtained, including miRNAs. Subsequently, unannotated reads were mapped in the 9311 genome (http://rice.genomics.org.cn/rice2/link/download.jsp) to analyze their genomic distribution using Bowtie. The mapped reads were aligned with known miRNAs from miRBase 21.0 (http://www.mirbase.org/). MiRDeep2 software (https://www.mdc-berlin.de/8551903/en/) was used to predict the novel miRNAs. The following parameters were applied to screen novel miRNAs [31–32]: (1) miRNA and miRNA* located in the opposite arms of stem-loop structure; (2) the minimal folding free energy indexes (MFEI) and index of MFEI of precursor below other structures; (3) the predicted miRNAs align with known miRNAs with a maximum of three mismatches allowed; (4) there is no loop or gap in miRNA/miRNA* complex; (5) miRNA/miRNA* with a maximum of four mismatches allowed.

To determine the statistics of the expression of miRNA, the normalized miRNA expression level was calculated via TPM [33]. Normalized expression (TPM) = the absolute readcount of the miRNAs/total count ofmiRNAs × 1,000,000. To detect the differentially expressed miRNAs, the expression level of the novel and known miRNAs between the PEG6000-free and PEG6000-treated samples was compared by using the log2-ratios and the False Discovery Rate (FDR). The absolute value of log2 Fold-Change (treatment/control) was greater than or equalto 1, and FDR was less than or equal to 0.01 as the standard.

### Expression analysis of the miRNA using stem-loop qRT-PCR

To confirm the miRNA expression data, stem-loop qRT-PCR was performed as described in Chen et al. [34]. Primers employed in the qRT-PCR are listed in S1 File. Typically, 200ng of RNA was digested by DNase I(Thermo scientific, USA) and reverse transcribed using a TaqManH MicroRNA Reverse Transcription Kit (Applied Biosystems, 4366596). Quantitative RT-PCR was performed on the ABI7500 Real-Time System (Applied Biosystems, USA) using EvaGreen 2×qPCR MasterMix-ROX(Abm, Canada). The reactions were incubated in a 96-well plate at 55°C for 2 min, 95°C for 10 min, followed by 40 cycles of 95°C for 30 s and 60°C for 1 min. All reactions were assayed in triplicate, and *OsActin* (GenBank Accession No. AB047313) was used as the reference gene. Normalized expression levels were calculated [35].

### RNA ligase-mediated 5′-rapid amplification of the cDNA ends (RLM-5′RACE)

Targets of the miRNAs were predicated using TargetFinder (http://www.acunetix.com/blog/docs/target-finder/) [36]. To identify the cleavage sites on the targets of the differentially expressed miRNAs, RLM-5′RACE was performed using the FirstChoiceH RLM-RACE Kit (Ambion, USA) according to the manufacturer’s instructions. The PCR products were cloned into the pEASY-T1(Transgen, China) vector for sequencing.

## Results

### Identification of small RNAs in the deep sequencing datasets

In this study, we constructed four small RNA libraries, called CL and CR from PEG6000-free samples and DL and DR from PEG6000-treated samples, where ‘R’ indicates the root tissue and ‘L’ indicates the shoot tissue. In total, 25921410, 21434119, 17573731, 20688030 raw reads were generated from CL, CR, DL, and DR, respectively. After removing the contents of the unknown baseswith≥10% reads, adaptor sequences, low quality sequences, and the sequences that were shorter than 18 nt or longer than 30 nt, we obtained 19657831, 13994393, 11037996, and 15570714 clean reads, respectively, which accounted for more than 60% of the raw reads (Table 1). By comparing the clean reads to the Silv, GtRNAdb, Rfam, and Repbase databases, the rRNA, tRNA, snRNA, snoRNA, and repeat sequences were removed. Furthermore, we obtained the unannotated reads, including miRNAs, which accounted for 60.37%, 59.72%, 67.14%, and 58.72% in the four datasets (Table 2). Moreover, the percentage of rRNA in the four datasets was < 40%, indicating that the four sRNA libraries were of a high quality. The numbers of unannotated reads that matched the genome included 11476361, 4697278, 5947313, and 6496665, accounting for 96.71%, 56.21%, 80.25%, and 71.06% in CL, CR, DL, and DR, respectively.

**Table 1:** The statistics of sequencing reads from four small RNA libraries in CWR.

Type	CL	CR	DL	DR
**Raw reads**	25921410	21434119	17573731	20688030
**Containing'N'reads**	716(0.00%)	10811(0.05%)	3350(0.02%)	4859(0.02%)
**Length <18**	1274949(4.92%)	2222601(10.37%)	645765(3.67%)	977311(4.72%)
**Length >30**	4987914(19.24%)	5206314(24.29%)	5886620(33.50%)	4135146(19.99%)
**Clean reads**	19657831(75.84%)	13994393(65.29%)	11037996(62.81%)	15570714(75.26%)
**Q30(%)**	93.85	90.15	94.63	94.88

The percentages indicates that different types of data accounts for the raw reads.

**Table 2:** The distribution of different small RNAs in four libraries.

Type	CL	CR	DL	DR
**Total small RNAs**	19657831	13994393	11037996	15570714
**rRNA**	6313423(32.12%)	4663983(33.33%)	2900772(26.28%)	4311029(27.69%)
**snRNA**	0(0.00%)	1(0.00%)	0(0.00%)	0(0.00%)
**snoRNA**	34945(0.18%)	21548(0.15%)	36701(0.33%)	38869(0.25%)
**tRNA**	1020808(5.19%)	790935(5.65%)	410211(3.72%)	1844937(11.85%)
**Repbase**	422029(2.15%)	160815(1.15%)	279191(2.53%)	233053(1.50%)
**Unannotated**	11866626(60.37%)	8357111(59.72%)	7411121(67.14%)	9142826(58.72%)
**Mapped Reads**	11476361	4697278	5947313	6496665
**Mapped reads(+)**	9982220	3526670	4895428	4477579
**Mapped reads(-)**	1494141	1170608	1051885	2019086

The percentages indicates that different types of data accounts for the total small RNAs.

**Fig 1:**
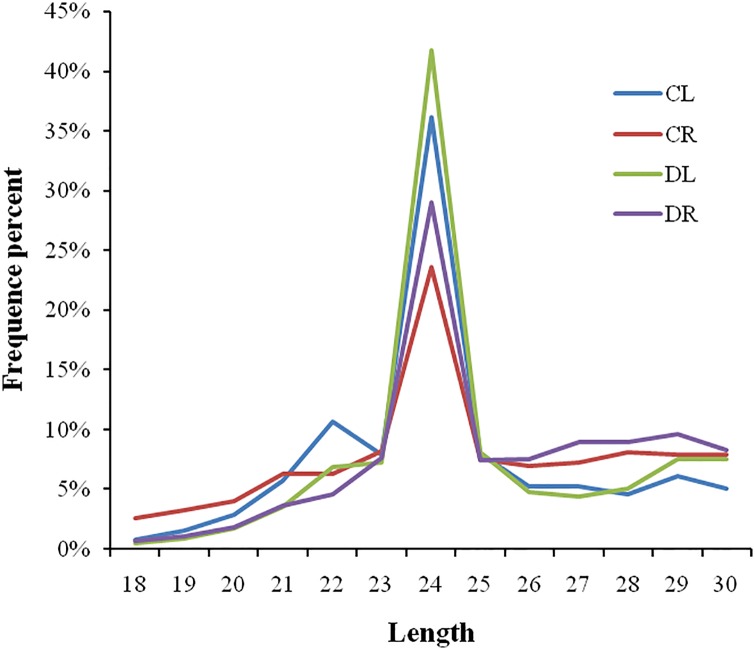
Length distribution of small RNAs in four libraries.

To investigate the small RNAs in the four libraries, the size distributions of the sRNAs ranged from 18 nt to 30 nt in length (Fig 1), and 24 nt sRNAs were dominant in all of the libraries, making up 36.18% (CL), 23.63% (CR), 41.77% (DL), and 29.03% (DR), respectively. Small RNAs are formed by DICER endonucleases before they act. Small RNA size classes were also decided by DCL1 (21 nt) and DCL3 (24 nt)[37]. Thus, this accounted for the majority of the 24 nt in our study. Interestingly, the percentage of 24 nt sRNAs were higher in the CL or DL, compared with the CR or DR. Furthermore, the content of the 24 nt sRNAs in the DL and DR were higher than that in the CL and CR samples.

### Differentially expressed miRNAs in the root and shoot in response to drought stress

In general, the transcriptional start sites of miRNA are located in intergenic regions, introns, and are reverse complementary to the coding sequence(CDS). Primary miRNAs (pri-miRNAs) are first transcribed from nuclear-encoded MIR genes and then cleaved by Dicer-like1 (DCL1), generating stem-loop miRNA:miRNA* duplexes known as pre-miRNAs. According to the biological characteristics of miRNAs, novel miRNAs can be predicted by miRDeep2 software. Compared with miRBase (Release 21: June 2014), we identified 540 mature miRNAs and 97 novel miRNAs from four libraries (Table 3). In total, 637 mature miRNAs were obtained.

**Table 3:** The distribution of miRNAs in four libraries.

Types	Known-miRNAs	Novel-miRNAs	Total
**CL**	487	97	584
**CR**	421	96	517
**DL**	456	97	553
**DR**	445	97	542
**Total**	540	97	637

**Fig 2:**
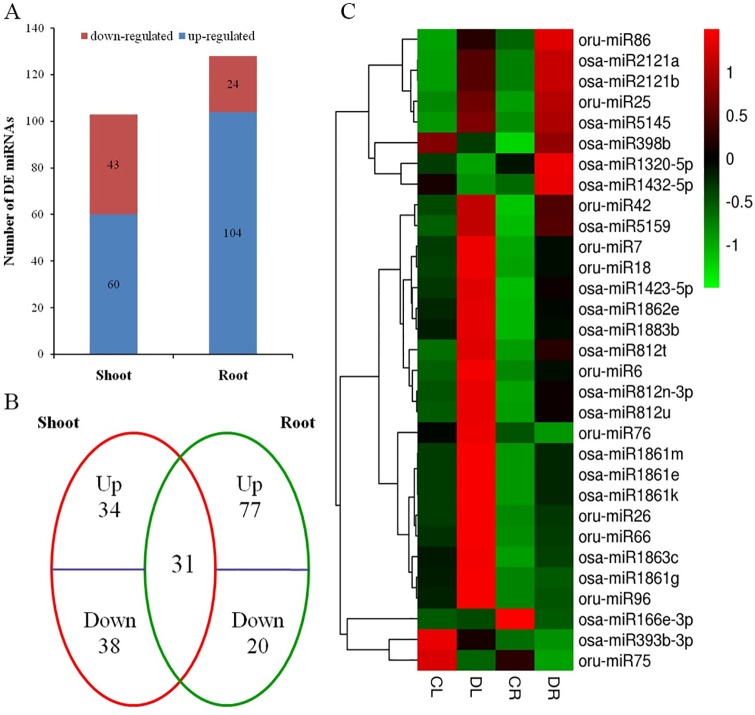
Differentially-expressed miRNA in the root and shoot. (A) The number of miRNAs up- or down-regulated by drought treatment; (B) A Venn diagrams showing the unique and shared miRNA in the CWR root and shoot under drought stress; (C) Hierarchical cluster analysis of 31 miRNAs that are regulated in both the shoot and root. The fold-change ratios of the miRNAs are indicated by the different colors.

After getting the miRNAs, the expression level was compared between the shoot (CL and DL) and root tissue (CR and DR). A total of 200 miRNAs were differentially expressed under drought stress. At the miRNA level, 77 known miRNAs that belong to 23 families, including 40 up-regulated and 37 down-regulated in the shoot (Fig 2A; S2 File), and 85 known miRNAs that belong to 46 families, including 65 up-regulated and 20 down-regulated in the root (Fig 2A; S3 File), were identified as differentially expressed. In addition, we predicted 26 new miRNA candidates from the shoot and 43 from the root that were differentially expressed during drought stress (Fig 2A; S2 and S3 Files). The Venn diagram (Fig 2B) showed that 31 miRNAs were common in the shoot and root, and 24 miRNAs were up-regulated, osa-miR393b-3p and oru-miR75 were down-regulated, whereas five miRNAs, i.e., oru-miR76, osa-miR1320-5p, osa-miR1432-5p, osa-miR166e-3p, and osa-miR398b, exhibited an opposite expression pattern in the shoot and root (Fig 2C).

**Fig 3:**
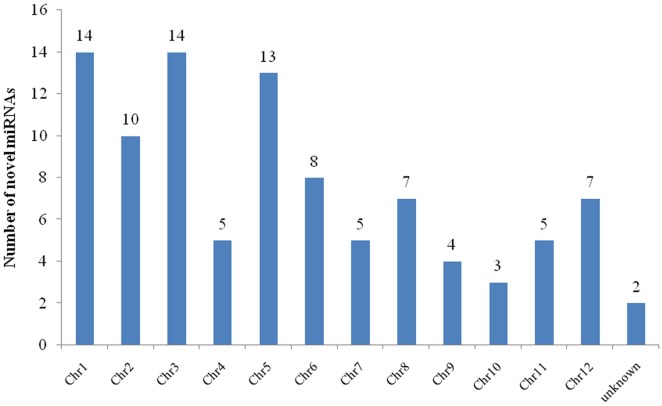
The distribution of novel miRNAs in chromosomes.

Besides the known miRNAs, we also identified 97 novel miRNAs (S4 File). All of the novel miRNAs were equally distributed in the 12 chromosomes of the rice genome, except oru-miR76 and oru-miR97, which were unknown (Fig 3). The precursors of oru-miR76 and oru-miR97 were mapped to scaffold008419_46931 and scaffold006334_46794 of the genome, respectively. Because the position of the scaffold was uncertain, the distribution of oru-miR76 and oru-miR97 on the chromosome was also unknown. The length of the 97 novel miRNAs ranged from 18 nt to 25 nt, and the majority were 24 nt. The average minimum free energy (MFE) value was -81.5.5 kcal mol^-1^ with a range of -205 kcal mol^-1^ to -39.9 kcal mol^-1^. A total of 11 novel miRNAs that existed in both the shoot and root were found to be differentially expressed in response to drought stress (Table 4; S5 File). Nine novel miRNAs were up-regulated both in the shoot and root, one was down-regulated, and another exhibited opposite expressions in the shoot and root.

**Table 4:** The novel drought-responsive miRNAs differently expressed in shoot and root.

miRNA ID	Chromosome	Arm	Mature sequence(5'-3')	L	MEFIs (kcal/mol)
oru-miR6	1	5p	aaaaaguuggaaguuuaugugugu	24	-93.20
oru-miR7	1	3p	augaauguggaaaaugcuagaaug	24	-101.80
oru-miR18	3	3p	uuuuuuuucuaggacagagggagu	24	-93.40
oru-miR25	3	3p	acuaaaggaccguagaauugcucu	24	-78.70
oru-miR26	4	5p	auaugaacgaaaaaaaccaauugc	24	-64.40
oru-miR42	6	3p	aauguggaaaaugcuagaaugacu	24	-53.90
oru-miR66	10	5p	auuuuccggcugaaagugacgagu	24	-93.40
oru-miR75	12	3p	accgcuucaugaacuuucagg	21	-58.60
oru-miR76	/	5p	auuagaaugagaaacauauacgug	24	-69.60
oru-miR86	2	3p	agauuuaaaucuaggauuggguuu	24	-73.40
oru-miR96	11	3p	gaggaccgcaugggaaaauaggcu	24	-85.00

### The tissue specificity characters of differentiated expressed miRNAs

**Fig 4:**
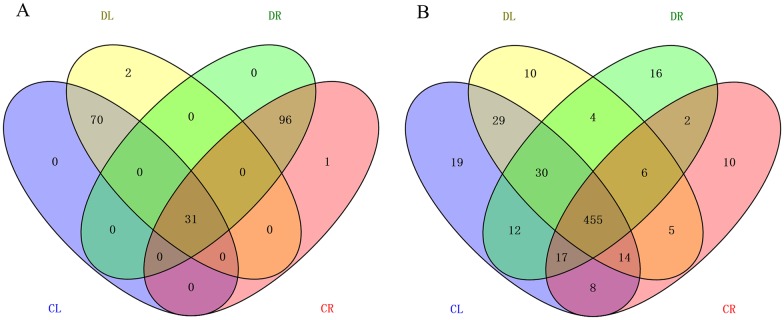
The Venn diagrams showing the overlaps among four libraries of miRNAs. (A) Differential miRNAs based on |log2 Fold-Change (treatment/control) |≥1 at FDR≤0.01; (B) miRNAs without being limited by the filter condition in the four libraries.

In order to identify whether the differentially expressed miRNAs have tissue specificity in the shoot or root, we analyzed the miRNAs using the Venn diagram (Fig 4A). The absolute value of log2 Fold-Change (treatment/control) was greater than or equalto 1, and FDR was less than or equal to 0.01 as the cut-off. In the shoot tissue, 70 miRNAs were differently expressed in response to drought stress, and 2 miRNAs were induced by drought stress. In the root, 96 miRNAs were differently expressed in response to drought stress, whereas one miRNA was specifically expressed in the CR. 31 miRNAs, which expressed differently in both the root and the shoot, as shown in Fig 2B.

Furthermore, we analyzed all of the miRNAs without being limited by the filter condition in the four libraries. The miRNA expression level was 0, which represented no expression, whereas greater than or equal to 0 indicated that expression existed. Finally, we obtained 58 and 28 miRNAs that specifically were expressed in the shoot and root. In total, 455 miRNAs existed both in the shoot and root in either the PEG6000-treated or PEG6000-free samples (Fig 4B). The expression level of the tissue-specific miRNAs provides a good foundation for understanding the development of tissues.

### qRT-PCR validation of the drought responsiveness of miRNAs

**Fig 5:**
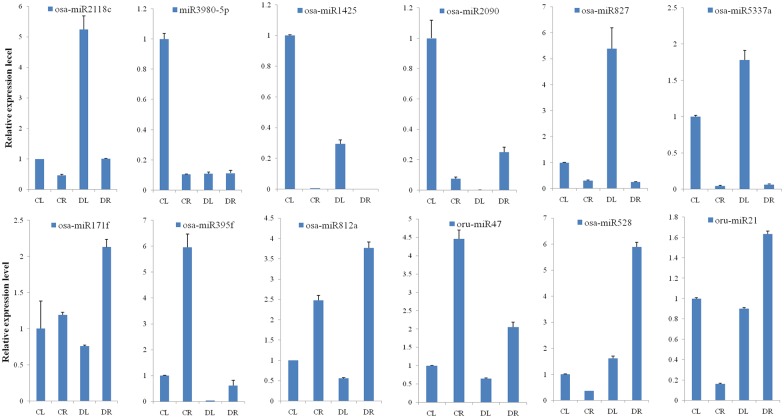
qRT-PCR validation of drought-responsive miRNAs in shoot and root. The expression values presented are the means of three technical replicates. *OsActin* was used as the reference gene.

To verify the reliability of the sequencing data, stem-loop quantitative RT-PCR was performed to validate the differentially expressed miRNAs. We chose 12 differentially expressed miRNAs that were expressed in response to drought from the shoot and root, including 2 novel miRNAs, oru-miR21 and oru-miR47. Meanwhile, we selected 2 known miRNAs (miR171f and miR395f) to insure the accuracy of qRT-PCR. First, miR171f and miR395f expressed in the root: miR171f was up-regulated, and miR395f was down-regulated (Fig 5). The results are consistent with reports and sequencing data [25], thus indicating the accuracy of the stem-loop qRT-PCR. Subsequently, miR2118c, miR3980, miR1425, miR2090, miR827, and miR5337a were present prior to expression in the shoot, and among them, miR2118c, miR827, miR5337a were up-regulated, and miR3980, miR1425, miR2090 were down-regulated in the shoot (Fig 5). Additionally, the expression level of the 6 miRNAs was low in the root due to drought response, which was in agreement with the sequencing data; however, there was some difference in the real data compared with the sequencing data. As shown in Fig 5, miR171f, miR812, miR528, and oru-miR21 expressed first in the root and were up-regulated, whereas miR171f and oru-miR21 still expressed in the shoot under drought stress. The miR395f and oru-miR47 were reduced in the root after drought stress, which was consistent with deep sequencing and indicated that the accuracy of the sequencing data was high.

### Target validation of the drought responsiveness of miRNAs

To identify the function of the miRNAs, target genes were predicted. The relationship between the miRNA and target genes was a negative correlation, and they mainly function as posttranscriptional regulators by targeting mRNAs via sequence complementarity, resulting in the degradation of targets [11–12]. Using the known and novel miRNAs under drought stress, targets of the miRNAs were predicted with TargetFinder (http://www.acunetix.com/blog/docs/target-finder/) [36]. In total, 498 rice genes were predicted to be potential targets of the 213 miRNAs. 88 miRNAs, that were drought responsiveness, were predicted with 197 targets (S6 File). Subsequently, we selected several miRNAs with a read count of >100 to verify the cleavage site by using RNA ligase-mediated 5’-rapid amplification of the cDNA ends (RLM-5′RACE).

**Fig 6:**
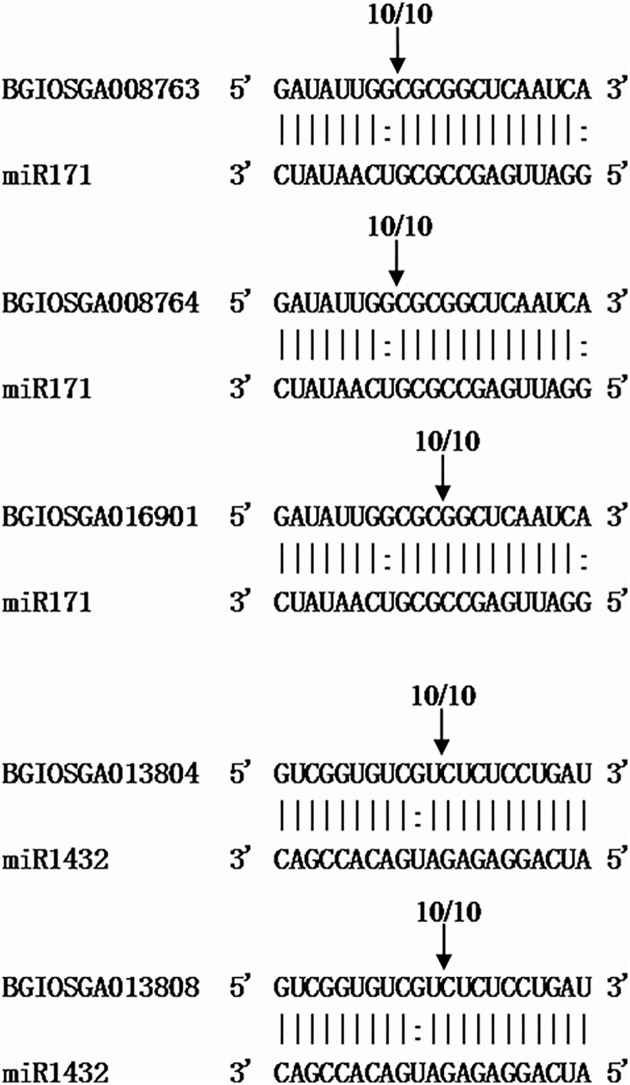
Verification of mRNA cleavage sites by 5’ RACE. 10/10 indicated the frequency of clones sequenced. The arrows indicate the cleavage sites.

As shown in Fig 6, miR171 had three target genes. The sequencing results of the 5’ RLM-RACE clones revealed that *BGIOSGA008763* and *BGIOSGA008764* mRNA were cleaved at the 5’ end at the 13^th^ to 14^th^ positions of the complementary site, whereas *BGIOSGA016901* was cleaved at the 10^th^ to 11^th^ positions. *BGIOSGA008763*, *BGIOSGA008764*, and *BGIOSGA016901* were transcript factors that belong to the GRAS domain-containing family. The three transcript factors are involved in the maintenance of the meristematic tissue and branch, the synthesis of chlorophyllose, and the development of the root. The targets of miR1432, *BGIOSGA013804* and *BGIOSGA013808*, were predicted to be EF-hand family proteins that participate in calcium ion-mediating ABA signaling pathways, and they are induced by drought and salinity [38]. Sequencing revealed that cleavage sites were located in the 10^th^to 11^th^ complementary region of miR1432 and in the targets (Fig 6). Generally, the perfect pairing at the 5’ end at the 9^th^ to 11^th^ position of complementarity is important for miRNA to cleave targets [9,13]. In fact, the cleaveage sites could be diverse. For instance, the cleavage site in the AK106348 mRNA by osa-miR3981-5p located at 13^th^ to 14^th^[39]; *Os09g03610* was cleaved by osa-miR819 at 12^th^ to 13^th^ and 16^th^ to 17^th^[23]. Furthermore, osa-miR1848 could regulate *OsCYP51G3* transcript, wherea the 5’ RLM-RACE test revealed that *OsCYP51G3* mRNA was cleaved downstream of the osa-miR1848/OsCYP51G3 mRNA complementary site [40]. Therefore, the targets of miRNAs need to be further verified.

## Discussion

### MicroRNAs involved in abiotic stress

Drought is the most important factor that limits rice production in drought-prone environments, especially at the reproductive stage, which causes varied degrees of spikelet sterility and poor grains [41–42]. Crop tolerance to drought is controlled by genetic and physiological regulation [43]. Many studies have shown that miRNAs participate in drought responsiveness in rice [25], *Arabidopsis* [44], *Triticumaestivum* [45], *Zea mays* [46], *Panicum virgatum* [47], *Populuseuphratica* [48], etc., indicating that miRNAs are involved in biotic and abiotic stress [14–15], [49]. In our study, high-throughput sequencing was performed to explore the miRNA expression profile in response to drought in CWR. Four libraries were constructed and sequenced with shoot and root tissues from 3-week-old CWR seedlings that were exposed to solutions with and without 16% PEG6000 for 24h. In total, 77 known miRNAs that belong to 23 families, including 40 up-regulated and 37 down-regulated in the shoot (Fig 2A;S2 File), and 85 known miRNAs that belong to 46 families, including 65 up-regulated and 20 down-regulated in the root (Fig 2A;S3 File), were identified as differentially expressed. In addition, we predicted 26 new miRNA candidates from the shoot and 43 from the root that were differentially expressed during drought stress.

Based on microarray data, the expression of 30 stress-regulated miRNAs were observed upon the withholding of water from the tillering to booting stage of rice [25]. Among them, 7 miRNAs, including miR156, miR168, miR169, miR171, miR319, miR396, and miR397 had the same expression pattern in our study. Interestingly, the expressions of miR171 and miR319 were not only increased, but also repressed during drought stress. It is probably that different degrees of drought stress lead to such differences [50]. On the other hand, high sensitivity of some miRNAs to subtle differences caused this difference in growing environments [51]. Furthermore, genome-wide high-throughput sequencing was performed on Dongxiang wild rice (*O*.*rufipogon* Griff.), and 17 up-regulated and 16 down-regulated miRNAs were investigated [4]. Nine miRNAs, including miR408, miR810, miR319, miR2863, miR444, miR166, miR167, miR818, and miR1861 were also detected in our study. Moreover, the expression patterns of miR319, miR166, miR810, and miR1861 were in agreement with our study [21]. In contrast to the Dongxiang CWR sequencing data, we explored miRNAs that were differentially expressed in shoot and root tissue under drought conditions, whereas whole seedlings that were exposed to air to mimic the drought treatment were used as the plant material in the Dongxiang CWR libraries [21]. As previously mentioned, *Oryza coarctata*isa halophyte, and the discovery of salt-responsive miRNAs was previously performed via high-throughput sequencing of the leaves [22]. Here, we report that differentially expressed miRNAs respond to drought in the shoot and root. All of the reports showed that miRNAs participate in the drought response in CWR.

In fact, rice production is not only impacted by drought but also the influence of saline and alkaline conditions [52], cold stress [53], heavy metal pollution [17], etc. The miR393, miR396 and miR820 responded to the saline and alkaline stress [52]. The miR156, miR164, miR166, miR167, miR169, miR171, and miR444 responded to Cd treatment in 7-day-old rice seedlings [17]. High throughput sequencing combined with computational analysis was used to survey the miRNAs from the seedlings of rice that underwent H_2_O_2_ treatments that resulted in oxidative stress. The expression profiles confirmed that miR169, miR397, miR528, miR1425, miR827, miR319a.2, and miR408-5p, which belong to 7 miRNAs families, were involved in H_2_O_2_ stress [39]. In combination with prior reports, we identified 13 miRNAs, including miR156, miR164, miR166, miR167, miR169, miR171, miR444, miR397, miR528, miR1425, miR827, miR319a.2, and miR408, that not only participated during drought stress but also responded to different stresses.

### Tissue-specific expression of miRNAs

The miRNA expression pattern has tissue specificity,except in the profile of the spatio-temporal expression. Plants must breathe just like animals. During the course of plant growth, the leaves can exchange air to promote plant growth via stoma and carry on photosynthesis and transpiration. It has reported that *Drought-Induced Wax Accumulation1* (*DWA1*) controls drought resistance by regulating the drought-induced cuticular wax deposition to improve the drought resistance of rice [54]. The miR528 is a conserved miRNA in monocots, and it has alow expression during rice leaf seeding, which has also been confirmed by our qRT-PCR experiments (Fig 5). With the growth of rice, the expression of miR528 increased in the leaves. It has been reported that overexpression of osa-miR528 in transgenic creeping bentgrass (*Agrostis stolonifera*) changed the plant development and improved the plant tolerance to salt stress and nitrogen (N) deficiency [55]. Morphologically, miR528-overexpressing transgenic plants display shortened internodes, increased tiller numbers, and upright growth [55]. Furthermore, overexpression of osa-miR528 in transgenic rice reduced the rice tolerance to Arsenic (As) stress, and the amino acid contents changed and the oxides increased both in the shoots and roots [56]. In our study, we investigated the drought resistance of rice during seedling stage, and the expression level of osa-miR528 was upregulated, especially in roots, indicating the improvement of drought resistance as well as report. A laccase-encoding gene, *LOC_Os01g62600*.*1*, that was previously reported to be one of the targets of miR528-5p, and the transcript levels increased during rosette leaves development in *Arabidopsis* [57–58]. It was functionally proven that miR528 was important for leaf development. Northern blotting and hybridization in situ show that osa-miR827 is strongly induced by phosphate (Pi) starvation in both the shoots and roots with signals concentrated in the mesophyll, epidermis, and ground tissues of the roots [59]. Meanwhile, the miR827 target, *OsSPX-MFS1*mRNA, down-regulated, whereas *OsSPX-MFS2* up-regulated during Pi starvation. Interestingly, both *OsSPX-MFS1* and *OsSPX-MFS2* were down-regulated in the overexpression of miR827 in transgenic rice [59]. According to qRT-PCR, upon drought stress, osa-miR827 was induced in DL but little expression in root tissues. In *Arabidopsis thaliana*, miR394 prior expressed in the overground portion whenever salt, drought, or ABA stress was present [60]. Combined with our sequencing data, miR528 and miR827 were up-regulated and expressed in the shoot first during drought treatment, which is consistent with other reports. In addition, miR171 up-regulated in response the drought stress in *Solanum tuberosum* [61]; mature miR171h molecules accumulated in the meristematic zone of the nodules, leading to a reduced mycorrhizal colonization and reduced nodule numbers in *Medicago truncatula* [62]. In this study, miR171 was also up-regulated during drought stress and was enriched in the roots. Meanwhile, the target of miR171 showed that miR171 maintained the development of the root, indicating that the function of miR171 needs to be further verified in regards to drought tolerance. At the miRNA level, 103 miRNAs, including 26 novel miRNAs, are differentially expressed between CL and DL in the shoot tissue (Fig 2A; S2 File). More functions of shoot-specific miRNAs could be detected that not only impact photosynthesis and transpiration but also influence rice production during abiotic stress.

The plant root system has various physiological functions, and the most important is water and nutrient uptake. The root transports to the overground portion after uptaking water and nutrient via the vascular system [63]. Furthermore, the plant uptakes water or more efficiently uses water in response to a need or to adapt to biotic or abiotic stress [64]. Hence, it is important to maintain the integrity of the root system for plant growth and production. Currently, next generation sequencing was performed to analyze the distribution of miRNAs between the root tips and the whole roots in rice. A total of 8 miRNAs were enrichment in the root tips, including miR169i-5p.2, miR390-3p, miR531b, miR1430, miR1847.2, miR1865-5p, miR1874-3p, and miR5082, whereas 24 miRNAs were identified that were significantly accumulated in the whole roots, including miR156, miR164, miR166, osa-miR169, miR171, miR393, miR408, miR528, miR529, osa-miR812, miR1320, osa-miR1432, miR1861, miR3979, etc.[65]. Bakhshi et al. investigated the miRNA drought responsiveness in rice roots, and 9 miRNAs, including miR156, miR159, miR168, miR171, miR396, miR528, miR529, miR1861, and miR3979, participated in drought stress, drought signaling, and wet signaling [66]. These results are consistent with our study regarding the differentially expressed miRNAs that are drought-responsive in the root tissue (S3 File). The miR167d targeted and negatively regulated auxin *OsARF12*. When *OsARF12* was knocked out, the expression of *OsMIR*, *OsIRT1*, and *OsSPR1* decreased, resulting in shortened roots, and decreased iron contents in the root tips [67]. In *Arabidopsis thaliana*, the same phenotype of miR169 was observed, indicating that miR169 plays a role in controlling root architecture [19]. Research shows that deep-root plants avoid drought stress by uptaking water from deep within the earth, and typical upland rice has a deeper root system than lowland rice [68]. As previously mentioned, the more deep the root system, more water is extracted from deep soil layers [69]. *DRO1*, a deep root gene, that was isolated from “Kinandang Patong”, has a higher yield than “IR64” [70]. *DRO1* was the first gene that was isolated from rice, and it was shown to be involved in the root system architecture that improves the tolerance of rice to drought.

## Conclusion

Four libraries were constructed and sequenced for shoot and root tissues from 3-week-old common wild rice seedlings that were exposed to solutions with and without 16% PEG6000 for 24h. In total, 77 known miRNAs that belong to 23 families, including 40 up-regulated and 37 down-regulated in the shoot (Fig 2A; S2 File), and 85 known miRNAs that belong to 46 families, including 65 up-regulated and 20 down-regulated in the root (Fig 2A; S3 File), were identified as differentially expressed. In addition, we predicted 26 new miRNA candidates from the shoot and 43 from the root that were differentially expressed during drought stress.

Furthermore, target genes of the differentially expressed miRNAs provided further evidence for their possible involvement in drought tolerance. These findings may provide new information for the identification of miRNAs in CWR (*O*.*rufipogon* Griff.) shoots and roots that respond to drought.

## Supporting Information

S1 File The primers used in this study. (XLSX)

S2 File The different expression of miRNAs between CL and DL libraries. (XLSX)

S3 File The different expression of miRNAs between CR and DR libraries. (XLSX)

S4 File Novel miRNAs in the common wild rice from four libraries. (XLSX)

S5 File The novel drought-responsive miRNAs differently expressed in shoot and root. (XLSX)

S6 File The miRNA targets involved in common wild rice drought stress. (XLSX)
